# Systematic integration of molecular and clinical approaches in HCV-induced hepatocellular carcinoma

**DOI:** 10.1186/s12967-024-04925-1

**Published:** 2024-03-12

**Authors:** Ciniso Sylvester Shabangu, Wen-Hsiu Su, Chia-Yang Li, Ming-Lung Yu, Chia-Yen Dai, Jee-Fu Huang, Wan-Long Chuang, Shu-Chi Wang

**Affiliations:** 1 Graduate Institute of Medicine, Kaohsiung Medical University, Kaohsiung, Taiwan; 2 Center for Liquid Biopsy and Cohort Research, Kaohsiung Medical University, Kaohsiung, 80708 Taiwan; 3 Present Address: Department of Medical Laboratory Science and Biotechnology, Kaohsiung Medical University, Kaohsiung, Taiwan; 4 Center of Excellence for Metabolic Associated Fatty Liver Disease, National Sun Yat-Sen University, Kaohsiung, Taiwan; 5 Faculty of Internal Medicine, School of Medicine, College of Medicine, Kaohsiung Medical University, Kaohsiung, 80708 Taiwan; 6 Hepatitis Research Center, Kaohsiung Medical University, Kaohsiung, Taiwan; 7 Hepatobiliary Division, Department of Internal Medicine, Kaohsiung Medical University Hospital, Kaohsiung Medical University, Kaohsiung, 80708 Taiwan; 8 Department of Medical Research, Kaohsiung Medical University Hospital, Kaohsiung, Taiwan

**Keywords:** HCV, miRNA, HCC, RCN1

## Abstract

**Background:**

MicroRNAs (miRNAs) play a crucial role in gene expression and regulation, with dysregulation of miRNA function linked to various diseases, including hepatitis C virus (HCV)-related hepatocellular carcinoma (HCC). There is still a gap in understanding the regulatory relationship between miRNAs and mRNAs in HCV-HCC. This study aimed to investigate the function and effects of persistent HCV-induced miRNA expression on gene regulation in HCC.

**Methods:**

MiRNA array data were used to identify differentially expressed miRNAs and their targets, and miRNAs were analyzed via DIANA for KEGG pathways, gene ontology (GO) functional enrichment, and Ingenuity Pathways Analysis (IPA) for hepatotoxicity, canonical pathways, associated network functions, and interactive networks.

**Results:**

Seventeen miRNAs in L-HCV and 9 miRNAs in S-HCV were differentially expressed, and 5 miRNAs in L-HCV and 5 miRNAs in S-HCV were significantly expressed in liver hepatocellular carcinoma (LIHC) tumors. Grouped miRNA survival analysis showed that L-HCV miRNAs were associated with survival in LIHC, and miRNA‒mRNA targets regulated viral carcinogenesis and cell cycle alteration through cancer pathways in LIHC. MiRNA-regulated RCN1 was suppressed through miRNA-oncogene interactions, and suppression of RCN1 inhibited invasion and migration in HCC.

**Conclusion:**

Persistent HCV infection induced the expression of miRNAs that act as tumor suppressors by inhibiting oncogenes in HCC. RCN1 was suppressed while miRNAs were upregulated, demonstrating an inverse relationship. Therefore, hsa-miR-215-5p, hsa-miR-10b-5p, hsa-let-7a-5p and their target RCN1 may be ideal biomarkers for monitoring HCV-HCC progression.

**Graphical Abstract:**

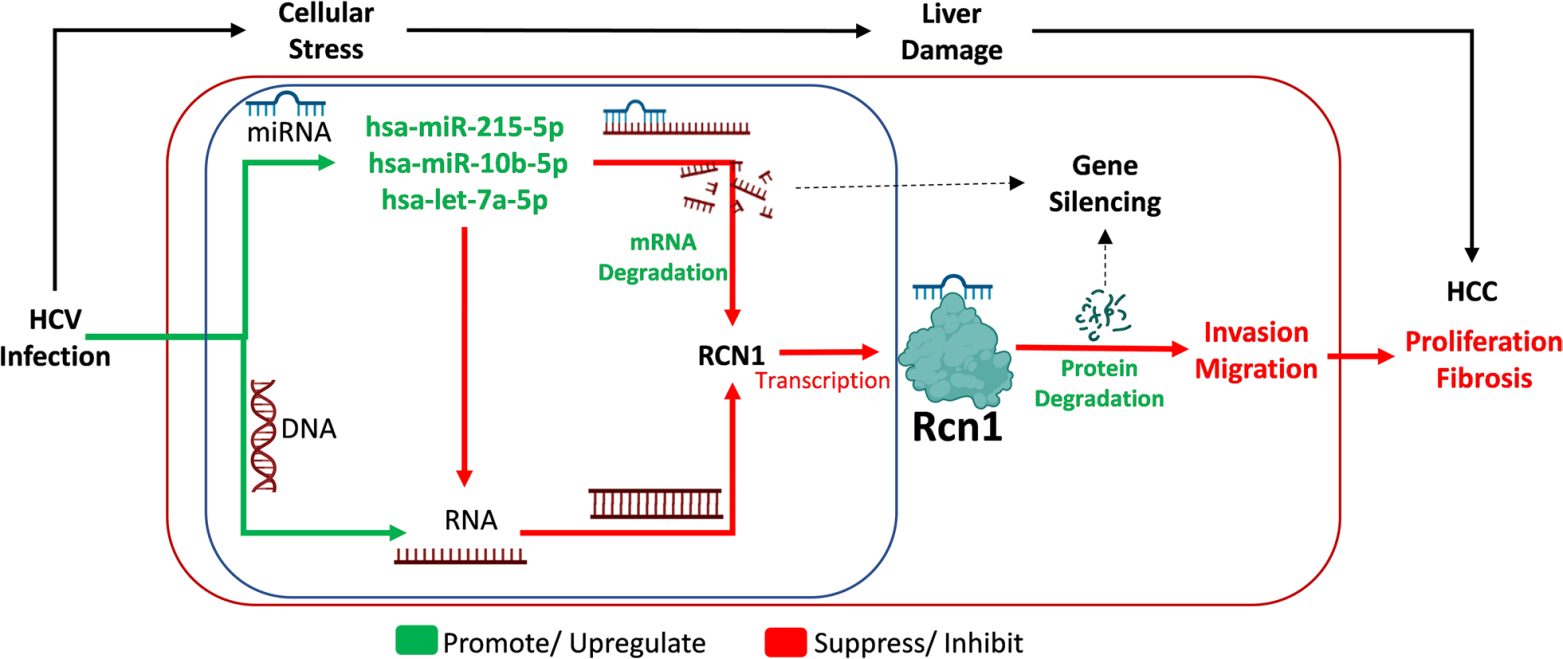

**Supplementary Information:**

The online version contains supplementary material available at 10.1186/s12967-024-04925-1.

## Background

Hepatitis C virus (HCV) infection is one of the etiologies significantly known to cause liver cirrhosis and hepatocellular carcinoma (HCC). A major concern is the estimated 170 million HCV chronically infected people and the toll it has on the public health system worldwide. The prevalence of HCV-related HCC ranges from 1–3% over a duration period of thirty years. A 15–20-fold increase has been observed in HCV patients at risk of HCC compared to HCV-negative patients in diverse studies [1]. Furthermore, clinical factors, including dual viral infection, alcohol use, stages of fibrosis and non-responsiveness to antivirals, have been associated with HCV-HCC [2–4]. HCV high viremia and duration of infection have been demonstrated to be significant risk factors for HCC, while virus treatment decreases the risk for HCC; however, it does not eradicate the risk of HCC. Recent studies have illustrated how HCV elimination effectively decreases the rate of liver cirrhosis [5] and HCC [6], leading to improved survival. HCV genetic heterogeneities directly regulate hepatocarcinogenesis, independent of liver fibrosis [4, 5]. These studies suggested that host genetics play a role in the effect and efficacy of anti-HCV therapy and HCV-related hepatocarcinogenesis [7].

MicroRNAs (miRNAs) are short noncoding RNAs (ncRNAs) of ~ 22 nucleotides that mediate gene expression through different functions, including gene silencing, which may lead to translational repression and degradation of mRNAs in HCC [8]. Therefore, miRNAs may regulate HCV-HCC genes. In our previous study, we demonstrated the oncogenetic pathway and genes in persistent HCV infection using next-generation mRNA sequences [9]. However, the miRNA‒mRNA regulatory interactions involved still need to be further studied, as there is currently limited information. In this study, a miRNA array was used to identify differentially expressed miRNAs in short-term and long-term HCV JFH1 replicon-infected Huh-7.5.1 cell line, and uninfected Huh-7.5.1 cell line was used as control to understand the effects of miRNA‒mRNA regulation in liver hepatocellular carcinoma (LIHC). Furthermore, HCV-HCC cohort expression data was integrated to understand the role of miRNAs in gene expression in HCV-HCC progression. The Graphical abstract illustrates our research.

## Materials and methods

### Cell culture and distinguishing intracellular HCV

The human hepatoma Huh-7.5.1 cell line was cultured and incubated in Dulbecco’s modified Eagle medium (DMEM) (Cat. # 21969035 Thermo Fisher Scientific, Waltham) mixed with 10% fetal bovine serum (FBS) (Lonza, Walkersville), 2% L-glutamate containing 200 mM solution in 0.85% NaCl solution (Lonza, Walkersville) and 2% antibiotics containing 10,000 U Pen./mL, 10,000 ug Strep./mL, 25 ug Amphotericin B/mL (Lonza, Walkersville) and 1% Non-Essential Amino Acids (NEAA) (Lonza, Walkersville) in a humidified 5% CO_2_/95% air atmosphere at 37 °C to reach 5 × 10^6^ cells in a 10 cm Petri dish. The Huh7.5.1 cells were infected via incubation with HCV JFH1-EYFP virus with for 6 h, after which the medium was replenished with fresh medium and cells were incubated for 5 days.

Huh-7.5.1 infected with JFH1 (HCV strain) cells were distinguished into high and low viral loads by fluorescence activated cell sorting (FACS) based on the amount of virus within the cells. After FACS, we reconfirmed the distinct populations by fluorescein isothiocyanate (FITC) mean fluorescence intensity (MFI). Low-viral-load cells were referred to as “early long-term HCV (eL-HCV)” and high-viral-load cells, referred to as “short-term HCV (S-HCV)”. To establish long-term infectious cells to represent persistent infection, eL-HCV cells from day 5 were cultured to day 30 and confirmed to be a distinct population of long-term-HCV (L-HCV) cells, referred to as long-term HCV (L-HCV). The methods are described in detail in our previously published studies [9, 10].

### MiRNA array

The samples used for miRNA expression profiling were short-term HCV (S-HCV, infected Huh-7.5.1 cell line) and long-term HCV (L-HCV, infected Huh-7.5.1 cells) and uninfected Huh-7.5.1 as a control group. Total RNA (100 ng) from each sample was used as initial input and was labeled with cyanine 3 (Cy3). Microarray slides after hybridization and washing were scanned using an Agilent Microarray Scanner (Agilent, Santa Clara, CA, USA). Scanning was performed at 5 μm resolution, and the dye channel was set to green (PMT5). Hybridization and labeling were performed according to the protocols in the Agilent miRNA microarray system. Microarray images were analyzed with Feature Extraction Software (Agilent). The signal after background subtraction was exported directly into GeneSpring GX10 software (Agilent) for quantile normalization. The mean of the normalized signal from three biological replicates was used for comparative expression analysis. The study data were uploaded to the Gene Expression Omnibus (GSE235959). From miRNA array data, 2549 miRNAs were identified, 533 miRNAs passed flags and normalization to the 75th percentile. A total of 123 miRNAs were identified with a raw ratio > 64. Finally, 73 miRNAs with a raw ratio > 3.2 were identified for further analysis in our study.

### The encyclopedia of RNA interactomes (ENCORI)

ENCORI is an online open-source platform for studying and analyzing miRNA- and miRNA-associated interactions. ENCORI further provides a platform to perform survival and differential expression analysis of miRNAs [11]. MiRNA expression in 370 cancer samples and 50 normal samples in LIHC were analyzed via ENCORI platform.

### OncomiR

OncomiR is an online platform for exploring miRNA dysregulation in cancer. The dysregulation of certain microRNAs (oncomirs) has been linked with specific cancer-forming (oncogenic) processes [12]. MiRNA survival outcomes in LIHC were analyzed by OncomiR.

### DIANA mirPath

DIANA-mirPath is a miRNA pathway analysis web server that provides accurate statistics while accommodating advanced pipelines. The interactions predicted and/or validated can be subsequently combined with advanced merging and meta-analysis functions [13]. MiRNA KEGG pathways were identified using DIANA.

### MiRNA-mRNA targets

MiRNAs target genes in a systematic manner and exhibit targetome specificity up to the pathway level, including KEGG. To identify miRNA‒mRNA targets, three different databases, miPathDB, TargetScan, and miRDB, were used to identify common genes.

### GO, pathway enrichment analysis

Gene Ontology (GO) term enrichment is a technique that allows interpretation of gene sets making use of the GO system of classification, in which genes are assigned to a set of predefined bins depending on their functional characteristics, including biological process (BP), molecular function (MF), cellular compartment (CC) and KEGG pathways. The 107 miRNA-mRNA targets identified in HCV-HCC cohort were analyzed for BP, MF, CC enrichment and the top enriched functions were identified to observe miRNA target mRNA.

### Patients cohorts

The initial screening phase included two randomly selected patients with HCV-related HCC (seropositive for HCV RNA) and three patients with non-B/non-C (NBNC, seronegative for HBV surface antigen [HBsAg] and antibodies to HCV [anti-HCV])-related HCC in Kaohsiung Medical University Hospital were randomly selected to explore potential hepatic RNAs relevant HCV related HCC. Hepatic RNA with differential expression were identified in paired T and adjacent non-tumor (ANT) tissue from surgical resection by using deep RNA sequencing. Four patients with HCV-related HCC and two patients with NBNC-related HCC who received tumor resection were recruited and were grouped in the validation set [14].

### Ingenuity pathways analysis (IPA)

IPA is a web-based bioinformatics application that allows data analysis results for functional analysis, associated network functions, canonical pathways and hepatotoxicity. Candidate miRNAs and their target genes were analyzed using IPA for interactive regulatory networks by merging their associated network functions.

### Kaplan‒Meier plotter

Kaplan‒Meier plotter is a web based analytic software which assesses the correlation between the expression of all genes (mRNA, miRNA, protein) and survival over 30 k + samples different tumor types, including liver cancer. Applied statistical tools include cox proportional hazards regression and the computation of the false discovery rate. The tool's primary purpose is the discovery and validation of survival biomarkers. Gene expression data and relapse-free and overall survival information is downloaded from different repositories including Gene Expression Omnibus (GEO), European Genome-Phenome Archive (EGA) and The Cancer Genome Atlas (TCGA) which is integrated and analyzed through the web-based platform. From the platform, mRNA sequence data from liver cancer patients (N = 362) were filtered by hepatitis virus to identify a total of 167 patients which were analyzed for disease specific survival. To analyze the prognostic value of a gene, the patient samples are split into two groups according to various quantile expressions of the proposed biomarker. The two patient cohorts were compared by a Kaplan‒Meier survival plot, and the hazard ratio with 95% confidence intervals and log-rank P value were calculated [15].

### mRNA-miRNA target sites

Sfold is a web server software which provides user-friendly statistical sampling paradigm for the prediction of RNA secondary structure. The software offers computational tools for the rational design of RNA-targeting nucleic acids, small interfering RNAs (siRNAs), antisense oligonucleotides and trans-cleaving ribozymes for gene knock-down research. Furthermore, modules of different RNAs offers comprehensive features for statistical representation of sampled structures. The results output are illustrated in graphical and text formats [16]. The mRNA-miRNA binding sites were identified using Sfold.

### Immunostaining

Uninfected Huh-7.5.1, and L-HCV cells were seeded and fixed using 100% ice-cold methanol (Sigma Aldrich, USA). For permeabilization, 0.1% Triton X-100 (Sigma Aldrich, USA) was used. For blocking, 2% BSA (Sigma Aldrich, USA) and primary antibody (anti-vimentin antibody [EPR3776], Abcam, USA) were used. Fluorescent dye–labeled secondary antibody with counterstain for the cytoskeleton and nucleus (DAPI), after which the target antigen was visualized by fluorescence microscopy.

### Real-time PCR

TRIzol reagent was used to isolate total RNA, of which 2 μg of RNA was used to synthesized cDNA was using a High-Capacity cDNA Reverse Transcription kit (Applied Biosystems, Thermo Fisher). For real-time PCRs, each sample was analyzed three times in a single 20 µL reaction using Power SYBR Green (Applied Biosystemes, Thermo Fisher) in an QuantStudio 5 Flex Real-Time PCR System (Thermo Fisher). Human sequence primers for RCN1 were as follows, forward primer: AACGGGTGCAGAAAAGATACA and reverse primer: AGGTAGTAACCATAGGTGGCTT and shRNA sequences for gene silencing were AGAAGCTAACTAAAGAGGAAA and CCGCAGAGTTTCATGATTCTT. Normalization of gene expression was compared to house-keeping gene GAPDH, which was calculated using threshold cycle (Ct) method using the Expression Suit Software (Thermo Fisher). Fold change expression was calculated using the 2^−(∆∆Ct)^ equation [9].

The sequence-specific primers for miRNA target: hsa-let-7a-5p, hsa-miR-10b-5p, and hsa-miR-215-5p. The expression of every miRNA was normalized to housekeeping- stably expressed hsa-miR-26a-5p. The sequence-specific forward primers based on miRbase (https://www.mirbase.org/cgi-bin/) were:

5ʹ-UGAGGUAGUAGGUUGUAUAGUU-3’ for hsa-let-7a-5p;

5ʹ -UACCCUGUAGAACCGAAUUUGUG-3’ for hsa-miR-10b-5p;

5ʹ-CUGACCUAUGAAUUGACAGCC-3’ for hsa-miR-215-5p; and.

5ʹ-UUCAAGUAAUCCAGGAUAGGC-3’ for hsa-miR-26a-5p.

The reagent for performing real-time qPCR miRNA was TaqMan Fast Advanced Master Mix (Applied Biosystems, Thermo Fisher), all primers set (hsa-let-7a-5p, hsa-miR-10b-5p, hsa-miR-215-5p, hsa-miR-26a-5p, and cDNAs) prepared in advance. CDNA synthesis was prepared using TaqMan Advanced miRNA Assay Kit (Cat. No: A25576, Applied Biosystems, Thermo Fisher). Reaction compositions consisted of 5 μl TaqMan Fast Advanced Master Mix, 1 μl PCR primer mix, 4 μl cDNA sample prepared in Bio-Rad C1000 Touch system which was diluted with a ratio of 1:40 before qPCR. Real time qPCR program QuantStudio 5 Flex Real-Time PCR System were set up as follows: Enzyme activation 95 °C for 20 s, Denature for 1 s at 95 °C and Anneal/ Extend at 60 °C for 20 s for 40 cycles, and melt curve analysis. The relative expression of the target gene was calculated by the relative quantification (ΔΔCt) using Ct value.

### Western blot

Total cellular protein was extracted using RIPA protein lysis buffer (cat. no. 89900, Thermo Fisher) with freshly added 1% protease inhibitor cocktail and 1 mM phenylmethylsulfonyl fluoride (PMSF) for 30 min on ice and then centrifuged at 13,000 × g for 20 min. A Bicinchoninic acid (BCA) assay protein kit (Beyotime, China) was used to detect protein concentration. A total of 10 μg of protein was determined using the Bio-Rad protein assay and used for Western blotting. After Western blotting, the samples were separated using 10% SDS-PAGE and transferred to PVDF membranes. PVDF membrane blots were blocked with 5% skim milk, followed by incubation with primary antibodies for E-cadherin (610182, BD Biosciences), N-cadherin (610920, BD Biosciences), Vimentin (550513, BD Biosciences), and actin (clone AC-40, A3853, Sigma-Aldrich) overnight at 4 °C in blocking buffer. Finally, the membrane blots were conjugated with horseradish peroxidase (HRP) secondary antibodies for 2 h and the signals were developed with chemiluminescence reagents (Amersham Biosciences). Chemiluminescence-associated bands were identified using ECL (Bio-Rad, ChemiDoc XRS + System) and quantified.

### Plasmid transfection

For gene overexpression experiments, L-HCV were transfected with the indicated gene overexprssion, using pCMV6 plasmid or empty vector using Lipofectamine 3000 (Invitrogen, Carlsbad, CA, USA) according to the manufacturer’s instructions [17]. The lentivirus was produced by transfection of Huh-7.5.1 cell line with control shRNA (shNC) or specific shRNAs targeting reticulocalbin 1 (*RCN1)* using Lipofectamine 3000. Viruses were collected after 48 h of transfection and purified using 0.45-μm filters. After infection with the indicated lentivirus in the presence of 8 μg/mL polybrene, L-HCV cells were selected with 1.5 μg/mL puromycin for 2 weeks. qRT‒PCR was used to detect the overexpression or knockdown efficiency.

### Transwell invasion assays

Transwell invasion assays were performed using transwells with polyethylene terephthalate membranes (24-well inserts, 8.0 μm, Corning) (Glendale, Arizona, USA) [18]. For the transwell invasion assay, 200 μL cell suspensions of L-HCV cells containing 5 × 10^5^ cells were loaded into the upper chamber of the transwell precoated with Matrigel (BD Biosciences, CA, USA). Next, 600 μL DMEM with 10% FBS was placed into the bottom of the well as a source of chemoattractants. Twenty-four hours later, the cells on the lower surface of the insert were fixed with methanol and stained with 0.5% crystal violet. The stained cells were visualized and photographed using a CKX41 microscope (Olympus, Japan). Five fields were randomly selected, and the cells were counted. Experiments were repeated three times, and the data are presented as the means ± SD.

### Wound healing assay

L-HCV were seeded in six-well plates (Corning) with 500 μm wound inserts and grown to confluence. The wounded area was recorded using an inverted phase-contrast microscope. The wound-healing rate was calculated as follows: ([wound width at 0 h − width at 48 h]/width at 0 h) [19].

### Statistical analysis

All statistical analyses were performed using GraphPad Prism software version 9.00 for Windows (GraphPad Prism Software, San Diego, CA, USA) and reported as the means ± standard deviation (SD). Student’s t test was used to evaluate the difference between two different groups. In addition, statistical significance between more than two groups was estimated by one-way analysis of variance (ANOVA). A value of P < 0.05 was deemed significant.

## Results

### L-HCV miRNAs regulate viral carcinogenesis and proteoglycans in LIHC

**Fig. 1:**
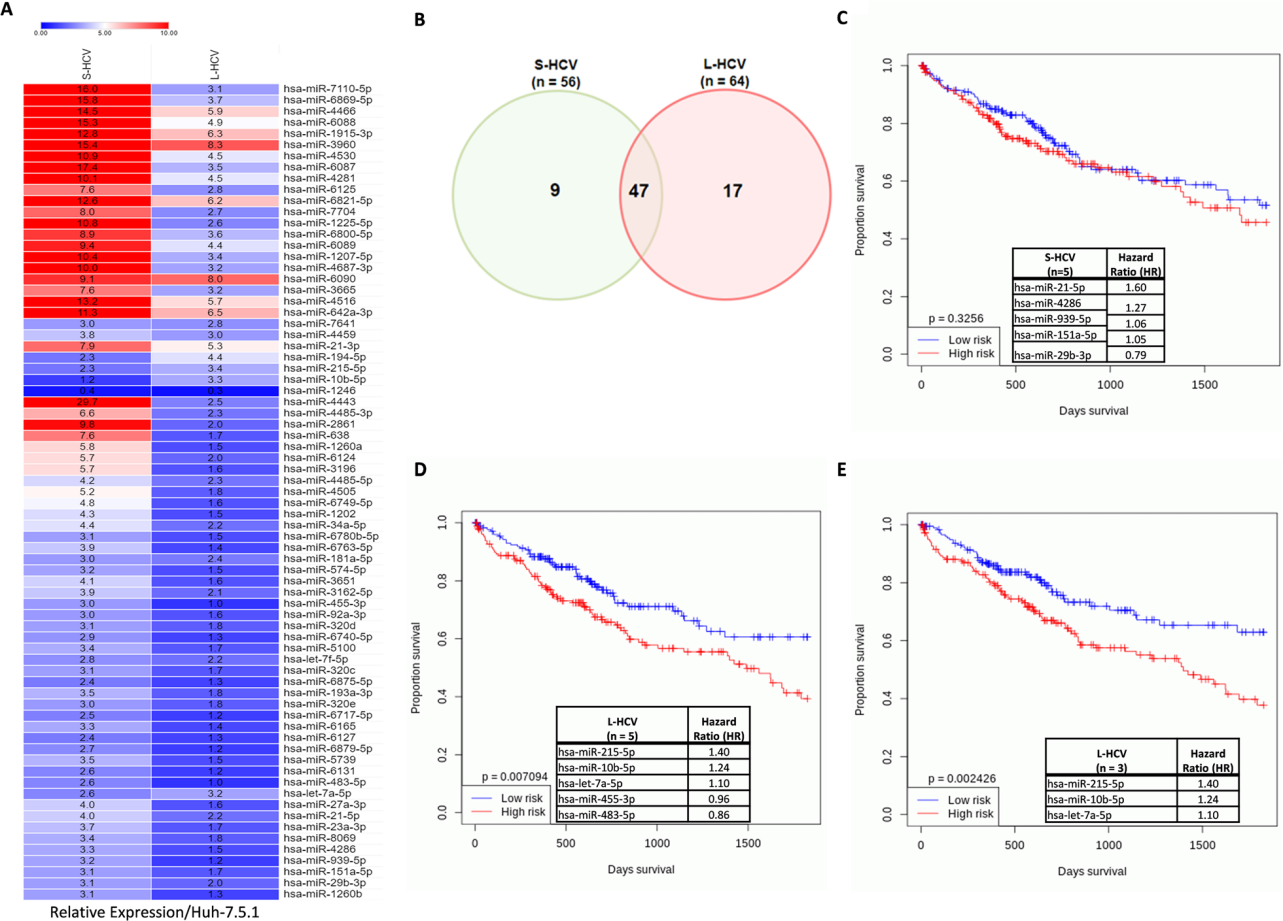
HCV differentially expressed miRNAs in LIHC. **A** A heatmap of miRNA expression in short-term HCV (S-HCV) and long-term HCV (L-HCV) relative raw data ratio fold change to uninfected Huh-7.5.1 cell line. **B** A Venn diagram of the number of miRNAs in S-HCV and L-HCV. Overall survival plot of grouped miRNAs in the LIHC cohort database: **C** S-HCV 5 miRNAs and (D) L-HCV 5 miRNAs. A total of 366 LIHC patients were stratified into 183 low-risk patients and 183 high-risk patients. **E** Overall survival plot of L-HCV 3 miRNAs with a hazard ratio (HR) > 1.0 in LIHC. Statistical significance p < 0.05*, p < 0.01**. paired t test

To investigate whether HCV infection altered the expression profile of miRNAs. MiRNAs with raw ratio greater than 3.2 and a significant p value (p < 0. 05) were filtered from miRNA array data as significantly expressed miRNAs. To examine the expression profile of 73 significantly expressed miRNAs, their relative raw ratio fold change was determined in S-HCV and L-HCV. MiRNA expression was altered by HCV infection (Fig. 1A). To identify differentially expressed (DE) miRNAs, 56 S-HCV significantly expressed miRNAs were compared to 64 L-HCV significantly expressed miRNAs. Nine DE miRNAs were identified only in S-HCV, 17 DE miRNAs were identified only in L-HCV, and 47 miRNAs overlapped between the S-HCV and L-HCV groups (Fig. 1B).

To determine clinically relevant miRNAs and emphasize the significance of miRNA candidates, DE miRNAs were analyzed between 370 cancer and 50 normal samples in LIHC by ENCORI platform which integrates pan cancer expression data of cancers from TCGA. A total of 5/9 S-HCV miRNAs (hsa-miR-21-5p, hsa-miR-4286, hsa-miR-939-5p, hsa-miR-151a-5p, and hsa-miR-29b-3p) (Additional file 1A–E) were significantly expressed (p < 0.01**) in LIHC, and 6/17 L-HCV miRNAs (hsa-miR-215-5p, hsa-miR-10b-5p, hsa-miR-455-3p, hsa-miR-6717-5p, hsa-miR-483-5p, and hsa-let-7a-5p (Additional file 1F–K) were significantly expressed in LIHC (highlighted in bold, Table 1). Furthermore, miRNAs showed altered expression between S-HCV and L-HCV (Additional file 1L).

**Table 1:** Differential Expressed S-HCV and L-HCV miRNAs expression in LIHC from ENCORI clinical database

S-HCV	LIHC	L-HCV	LIHC
(n = 9)	P-Value	(n = 17)	P-Value
hsa-miR-27a-3p	0.012	hsa-miR-194-5p	0.12
**hsa-miR-21-5p**	**2.60E-34**	**hsa-miR-215-5p**	**3.40E-05**
hsa-miR-23a-3p	0.17	**hsa-miR-10b-5p**	**1.40E-22**
hsa-miR-8069	0.71	hsa-miR-181a-5p	0.044
**hsa-miR-4286**	**1.80E-06**	**hsa-miR-455-3p**	**1.40E-16**
**hsa-miR-939-5p**	**0.001**	hsa-miR-92a-3p	0.1
**hsa-miR-151a-5p**	**3.00E-06**	hsa-miR-6740-5p	0.72
**hsa-miR-29b-3p**	**0.0085**	hsa-miR-6875-5p	0.15
hsa-miR-1260b	0.27	hsa-miR-320e	0.014
		**hsa-miR-6717-5p**	**0.00087**
		hsa-miR-6127	0.71
		hsa-miR-6879-5p	0.71
		hsa-miR-6131	0.71
		**hsa-miR-483-5p**	**5.30E-07**
		**hsa-let-7a-5p**	**0.00033**
		hsa-miR-7641	0.69
		hsa-let-7f-5p	0.094

Highlighted in bold are miRNAs significant by P < 0.01

L-HCV 5 miRNAs grouped overall survival analysis by Oncomir showed that L-HCV miRNAs were associated with survival (p = 0.007) in LIHC, whereas S-HCV 5 miRNAs grouped overall survival analysis showed that S-HCV miRNAs were not associated with survival (p = 0.326) in LIHC (Fig. 1C and D). L-HCV hsa-miR-6717-5p was excluded from further analysis due to missing survival data in LIHC. S-HCV miRNAs were excluded from further investigation.

To further improve the significance of identifying miRNA candidates, a hazard ratio (HR) > 1.0 of L-HCV 5 miRNAs was considered (Fig. 1D). Hazard ratios are measures of association widely used in prospective studies by comparing the hazard function among those exposed to the hazard function among non-exposed individuals [20]. The hazard ratio can be considered an estimate of relative risk, which is the risk of an event (or of developing a disease) relative to exposure [21]. Each estimated hazard ratio has a corresponding confidence interval and *P* value [22]. A hazard ratio > 1 suggests an increased risk, and a hazard ratio < 1 suggests a decreased risk. A hazard ratio of 1 suggests no difference in the risk of LIHC patient survival [21]. Three (hsa-miR-215-5p, hsa-miR-10b-5p, hsa-let-7a-5p) L-HCV miRNAs distinctly distinguished their effect on survival between low and high-risk group in LIHC (Fig. 1E). In contrast, miRNAs (hsa-miR-455-3p, hsa-miR-483-5p) with HR < 1.0 weren’t associated (p = 0.1) with overall survival (Additional file 1M).

Furthermore, there was no survival association of the grouped expression of these miRNAs (hsa-miR-215-5p, hsa-miR-10b-5p, hsa-let-7a-5p) in other cancers, including ovarian serous systadenocarcinoma (OV), esophageal carcinoma (EASCA), colon adenocarcinoma (COAD), lung adenocarcinoma (LUAD), pancreatic adenocarcinoma (PAAD), and prostate adenocarcinoma (PRAD) (Additional file 2**)**. The results confirmed that the combined expression of L-HCV miRNAs was associated with overall survival in LIHC.

To investigate the key function of L-HCV miRNAs with an HR > 1.0, KEGG analysis showed that the more interesting top miRNA pathways were associated with the cell cycle (p = 1.69E-09, miRNAs = 3), viral carcinogenesis (p = 5.10E-08, miRNAs = 3), proteoglycans in cancer (p = 1.49E-04, miRNAs = 3), p53 signaling pathway (p = 1.13E-03, miRNAs = 3), and pathways in cancer (p = 4.73E-03, miRNAs = 3). The results showed that miRNAs are implicated in cancer pathways (most enriched category), including viral carcinogenesis and proteoglycans in cancer, suggesting that indeed these miRNAs (hsa-miR-215-5p, hsa-miR-10b-5p, hsa-let-7a-5p) were HCV induced and linked to LIHC.

In summary, the findings suggest that HCV infection induces the expression of miRNAs that regulate viral carcinogenesis and proteoglycans involved in remodeling of tumor stroma in LIHC.

### HCV miRNAs target genes altered extracellular matrix in response to stimuli by regulating fibrillar collagen in liver damage in HCC

**Fig. 2:**
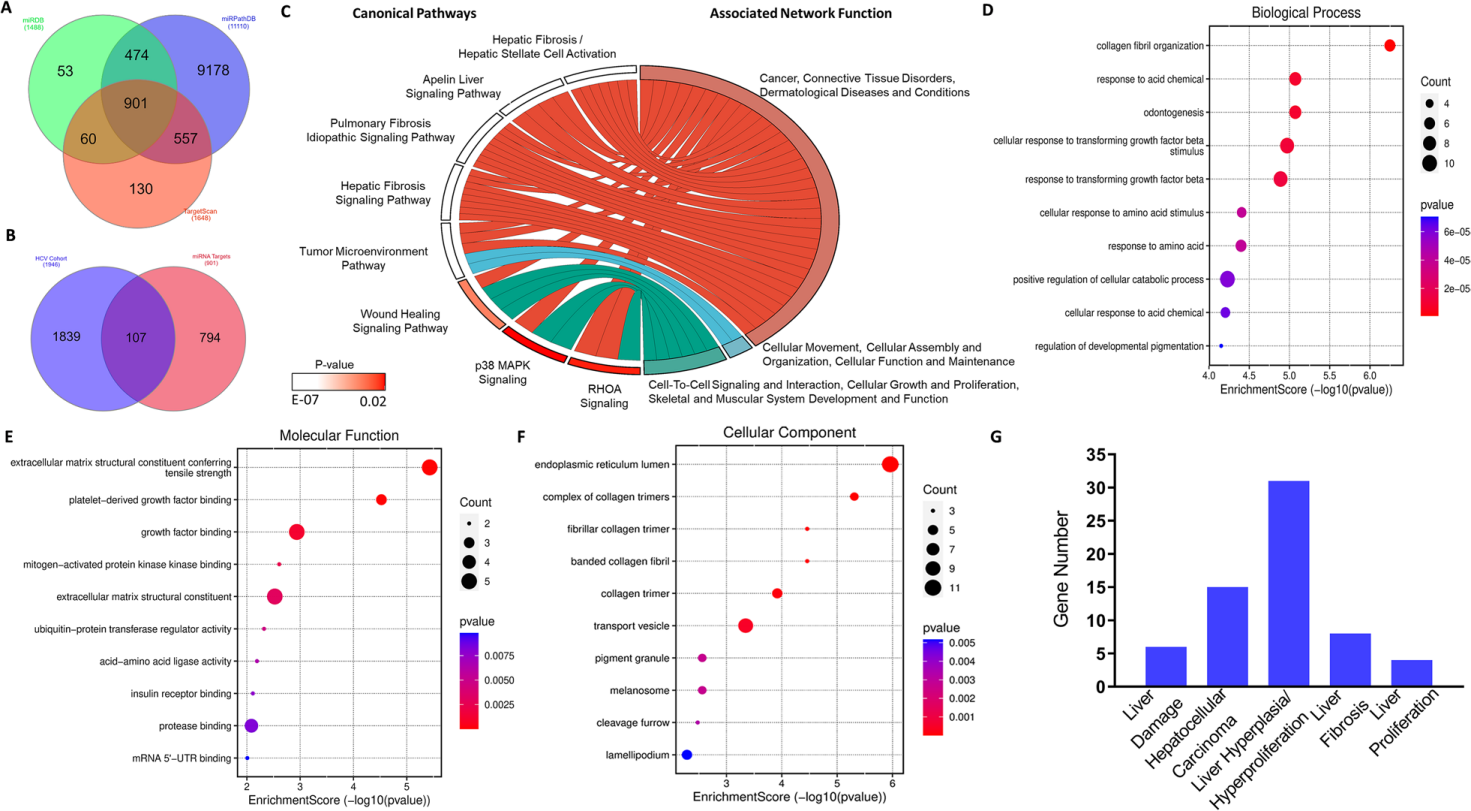
MiRNA-mRNA functional analysis and hepatotoxicity. **A** A Venn diagram showing miRNA‒mRNA targets: L-HCV miRNA (hsa-miR-215-5p, hsamiR-10b-5p, hsa-let-7a-5p) targets from miRDB (n = 1488), TargetScan (n = 1648) and miRPathDB (n = 11,110) and their intersecting miRNA‒mRNA targets (n = 901). **B** A Venn diagram showing genes in the HCV-HCC cohort (n = 1946) and miRNA‒mRNA targets (n = 901) with their intersecting genes (n = 107). **C** Canonical pathways and associated network functions of the combined overlapping (n = 107) genes in the HCV-HCC cohort. Gene Ontology (GO) enrichment analysis: **D** biological process, **E** molecular function and **F** cellular component. **G** Ingenuity Pathways Analysis (IPA) hepatotoxicity of 107 miRNAs‒mRNA predicted targets. Statistical significance value P < 0.05

To investigate the effects of miRNA expression on gene expression in the HCV-HCC cohort, miRNA‒mRNA targets were identified and analyzed for gene ontology functional enrichment. A total of 901 intersecting miRNA‒mRNA targets were identified from three miRNA target databases (miRDB, TargetScan, miRPathDB) for the three miRNA targets (hsa-miR-215-5p, hsa-miR-10b-5p, hsa-let-7a-5p) (Fig. 2A).

To identify miRNA targets from an HCV-HCC cohort, HCV-HCC expression data were identified from comparative RNA sequencing of tumor and adjacent non-tumour tissues in our previously published studies [23, 24]. Gene expression data in the Gene Expression Omnibus (GEO-GSE154211) of the HCV-HCC cohort were accessed to identify 1946 genes by comparing HCV-HCC genes to non-HCV/ non-HBV (NBNC) and HBV expressed mRNAs to eliminate non-specific genes. The 1946 genes were compared to the 901 miRNA targets to identify 107 overlapping genes (Fig. 2B).

The 107 miRNA targets in the HCV-HCC were analyzed for canonical pathways and associated network functions. Associated network functions showed that miRNA targets were clustered into ‘Cancer, Connective Tissue Disorders, Dermatological Diseases and Conditions’ (Focus Molecules = 24, Score = 61), ‘Cellular Movement, Cellular Assembly and Organization, Cellular Function and Maintenance’ (Focus Molecules = 16, Score = 36), and ‘Cell-To-Cell Signaling and Interaction, Cellular Growth and Proliferation, Skeletal and Muscular System Development and Function’ (Focus Molecules = 20, Score = 20) (Fig. 2C). Genes identified from network clusters were further linked to their canonical pathways, which were hepatic fibrosis/hepatic stellate cell activation (p = 1.62E-07), apelin liver signaling (p = 2.95E-07), pulmonary fibrosis idiopathic signaling (p = 3.77E-07), hepatic fibrosis signaling (p = 2.65E-06), tumor microenvironment (p = 3.71E-05), wound healing signaling (p = 1.59E-02), p38 MAPK signaling (p = 2.50E-02), and RHOA signaling (p = 2.42E-02) (Fig. 2C). MiRNA target gene networks regulate cancer, cellular movement and cell-to-cell interactions to alter tumor microenvironment signaling.

To investigate the mechanism by which miRNAs alter tumor microenvironment signaling pathways, miRNA targets in the associated network functions were analyzed for functional enrichment. Biological process (BP) enrichment showed that miRNA targets were involved in collagen fibril organization (genes = 6, p = 5.650E-07), response to acid chemical (genes = 7, p = 8.46E-06), odontogenesis (genes = 7, p = 8.46E-06), cellular response to transforming growth factor beta stimulus (genes = 9, p = 1.07E-05), response to transforming growth factor beta (genes = 9, p = 1.29E-05), cellular response to amino acid stimulus (genes = 5, p = 3.92E-05), response to amino acid (genes = 6, p = 4.00E-05), positive regulation of cellular catabolic process (genes = 10, p = 5.89E-05), cellular response to acid chemical (genes = 5, p = 6.26E-05), and regulation of developmental pigmentation (genes = 3, p = 7.06E-05) (Fig. 2D). MiRNA targets facilitate the cellular response to stimuli (highest count in BP), such as transforming growth factor.

Molecular function (MF) enrichment showed that miRNA targets were involved in extracellular matrix structural constituent conferring tensile strength (genes = 5, p = 3.71E-06), platelet-derived growth factor binding (genes = 3, p = 2.99E-05), growth factor binding (genes = 5, p = 1.16E-03), mitogen-activated protein kinase binding (genes = 2, p = 2.47E-03), extracellular matrix structural constituent (genes = 5,p = 2.99E-03), ubiquitin-protein transferase regulator activity (genes = 2, p = 4.76E-03), acid-amino acid ligase activity (genes = 2, p = 6.46E-03), insulin receptor binding (genes = 2, p = 7.73E-03), protease binding (genes = 4, p = 8.21E-03), and mRNA 5'-UTR binding (genes = 2, p = 9.81E-03) (Fig. 2E). MiRNA targets bind to growth factors (highest gene count) and maintain the extracellular matrix (top enriched, highest gene count).

Cellular component (CC) enrichment of miRNA targets showed that they were localized in the endoplasmic reticulum lumen (genes = 11, p = 1.10E-06), complex of collagen trimers (genes = 4, p = 4.89E-06), fibrillar collagen trimer (genes = 3, p = 3.47E-05), banded collagen fibril (genes = 3, p = 3.47E-05), collagen trimer (genes = 5, p = 1.21E-04), transport vesicle (genes = 9, p = 4.50E-04), melanosome (genes = 4, p = 2.74E-03), pigment granule (genes = 4, p = 2.74E-03), cleavage furrow (genes = 3, p = 3.31E-03), and lamellipodium (genes = 5, p = 5.17E-03) (Fig. 2F). MiRNA targets were localized in the endoplasmic reticulum lumen (highest gene count) in fibrillar collagen components (highest criteria).

Furthermore, miRNA targets were analyzed for their effects on the liver. The results revealed that miRNA targets altered liver damage (genes = 6, p = 4.59E-04—2.56E-05), liver fibrosis (genes = 8, p = 2.40E-02—2.70E-04), liver proliferation (genes = 4, p = 6.41E-02—2.70E-04), liver hyperplasia/hyperproliferation (genes = 29, p = 2.16E-01—4.75E-04), and liver necrosis/cell death (genes = 5, p = 4.84E-02—6.45E-04) (Fig. 2F). MiRNA targets are involved in liver damage and fibrosis in HCC.

In summary, miRNA targets in response to stimuli such as HCV infection altered extracellular matrix constituents through fibrillar collagen to regulate liver damage in HCC.

### HCV miRNAs target RCN1-regulated EMT in HCV-HCC

**Fig. 3:**
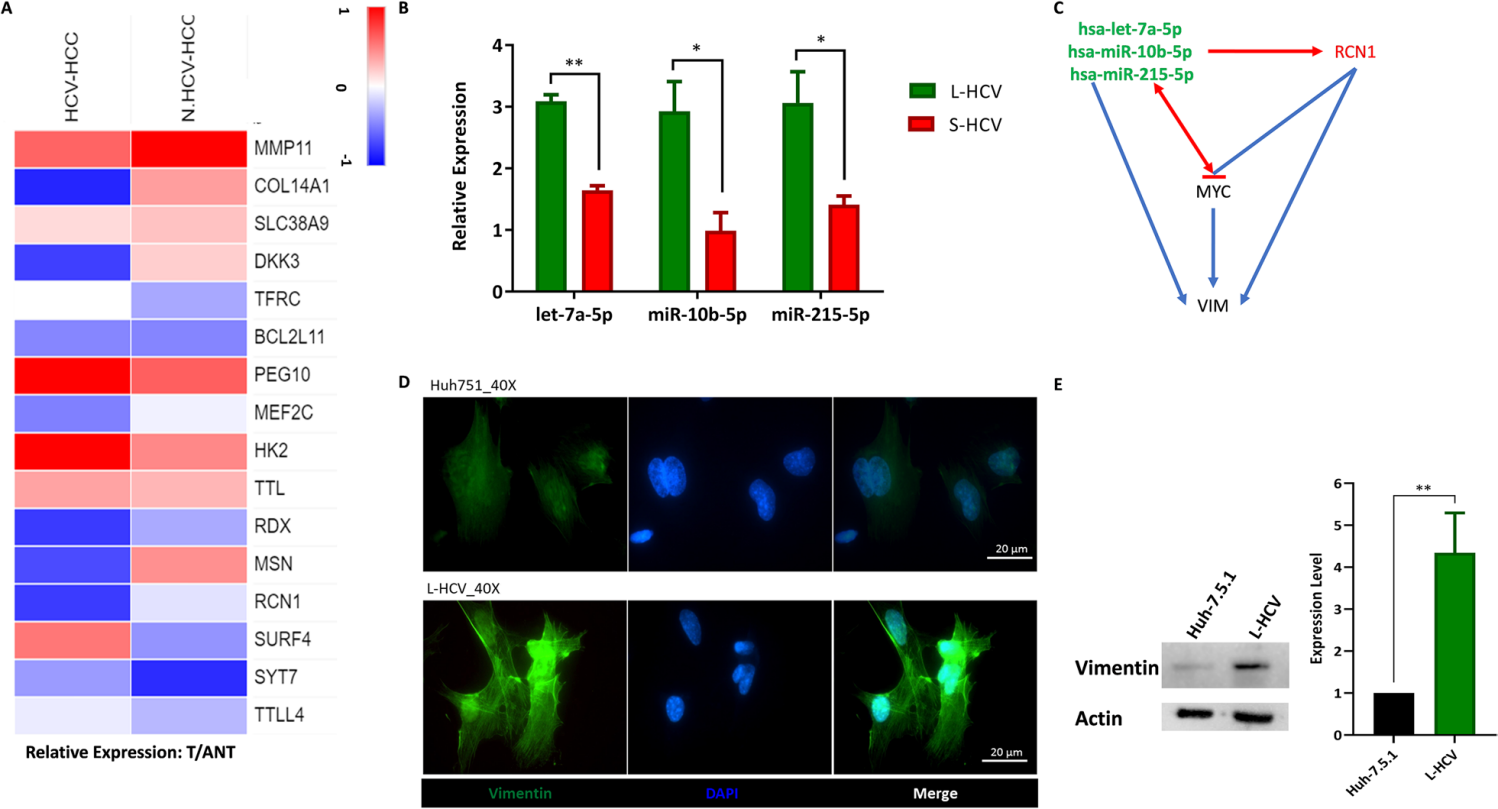
MiRNAs target interactive networks in regulating HCV-HCC. **A** Heatmap of disease-specific survival-associated miRNA target expression between tumor (T) and adjacent non-tumor (ANT) samples in the HCV-HCC cohort **B** Relative expression of L-HCV miRNAs (hsa-miR-215-5p, hsa-miR-10b-5p, hsa-let-7a-5p) compared to S-HCV. **C** An interactive network of miRNAs, miRNA-target RCN1, oncogene MYC and EMT marker Vimentin (VIM). **D** ICC staining imaging at 40X magnification of EMT marker Vimentin (VIM) expression in Huh-7.5.1 and L-HCV cells. DAPI (4’,6-diamidino-2-phenylindole): Cell Nuclear Staining. Merged images of VIM expression and cells. Experiments were conducted in triplicates. **E** Expression of VIM between Huh-7.5.1 and L-HCV. Statistical significance p < 0.05*, p < 0.01**. paired t test

To investigate the effects of the expression of miRNA targets in HCV-HCC, miRNA targets in gene ontology enrichment were analyzed for disease-specific survival (DSS) in hepatitis-related liver cancer. Additional file 3 shows sixteen miRNA targets (A) RCN1, (B) MMP11, (C) COL14A1, (D) SLC38A9, (E) DKK3, (F) TTLL4, (G) SURF4, (H) TFRC, (I) BCL2L11, (J) PEG10, (K) MEF2C, (L) SYT7, (M) HK2, (N) TTL, (O) RDX, and (P) MSN that were significantly associated with survival in hepatitis-related liver cancer. The expression of these genes was altered between tumor and adjacent non-tumor samples in HCV-HCC patients and non-HCV-HCC patients (Fig. 3A). Furthermore, hsa-let-7a-5p, hsa-miR-10b-5p, and hsa-miR-215-5p were upregulated in L-HCV compared to S-HCV, (p = 0.0038, p = 0.0403, p = 0.0473), respectively (Fig. 3B). The expression of miRNAs and their target genes was linked to HCV infection.

To identify and investigate the role of candidate miRNA‒mRNA targets in HCV-HCC, an interactive network was constructed using DSS genes. The results showed that RCN1 is a direct regulator of the DSS gene network, while other genes interact with this network via intermediate genes. Therefore, RCN1 was identified as a candidate miRNA‒mRNA target. MiRNA binding sites to RCN1 were confirmed using Sfold analysis by identifying two highly conserved regions in miRNAs that bind to RCN1. MiRNA seed (nucleotides 2–7), particularly those in 3′ untranslated regions (3′UTRs), are conserved and coding sequences are mostly conserved regions within a gene and the presence of miRNA binding sites suppress the accumulation of mutations [25]. Hsa-let-7a-5p binds to RCN1 region with start codon (AUG) (Additional file 4A, B), hsa-miR-10b-5p binds to start codon (AUG) and stop codon (UAA) region in RCN1 (Additional file 4C, D), and hsa-miR-215-5p binds to stop codon (UAG) in RCN1 region (Additional file 4E, F). This data further assisted in confirming a regulatory relationship between miRNA and RCN1.

Evidence has shown RCN1, endoplasmic reticulum protein, has been demonstrated to induces drug resistance and malignancy in HCC by activating c-MYC signaling [26]. Thus, we integrated c-MYC in the interactive network construction to understand the relationship between RCN1 and oncogenes such as c-MYC. Vimentin has been shown to bind to focal adhesions and fibrillar collagen to stabilize cell‒matrix adhesions that are subjected to shear stress leading irregular, less distinct and less mechanically stable cells [27, 28]. Furthermore, vimentin has different effects on host cells during invasion by specific viruses that express on their surface ligands for vimentin such as HCV [27, 29].Therefore, we also integrated vimentin in the interactive network analysis to understand how the extracellular matrix (ECM) and fibrillar collagen in extracellular matrix may be altered during HCV infection.

The interactive network results showed that miRNAs and RCN1 regulated VIM. Furthermore, miRNAs (hsa-miR-215-5p and hsa-miR-10b-5p, let-7a-5p) interacted with HCV-HCC genes via RCN1 and VIM (Fig. 3C). Finally, we constructed a summative figure to illustrate our findings (Fig. 3C).

Based on the HCV-HCC cohort expression data, the results showed that RCN1 was suppressed in HCV-HCC (Fig. 3A). Combined, the results suggest that the upregulation of the three L-HCV miRNAs (hsa-miR-215-5p, hsa-miR-10b-5p, hsa-let-7a-5p) led to the downregulation of RCN1 (Fig. 3C). The results showed that L-HCV 3-miRNAs suppressed the oncogene MYC. Furthermore, the EMT marker vimentin (VIM) was upregulated (P = 0.0037) in L-HCV cells compared to Huh-7.5.1 cell line (Fig. 3D and E), suggesting that RCN1 may regulate EMT in HCV-HCC. Collectively, the findings suggest that HCV miRNAs target RCN1-regulated EMT in the HCV-HCC cohort.

### RCN1 acts as a tumor suppressor by inhibiting invasion and migration in HCV-HCC

**Fig. 4:**
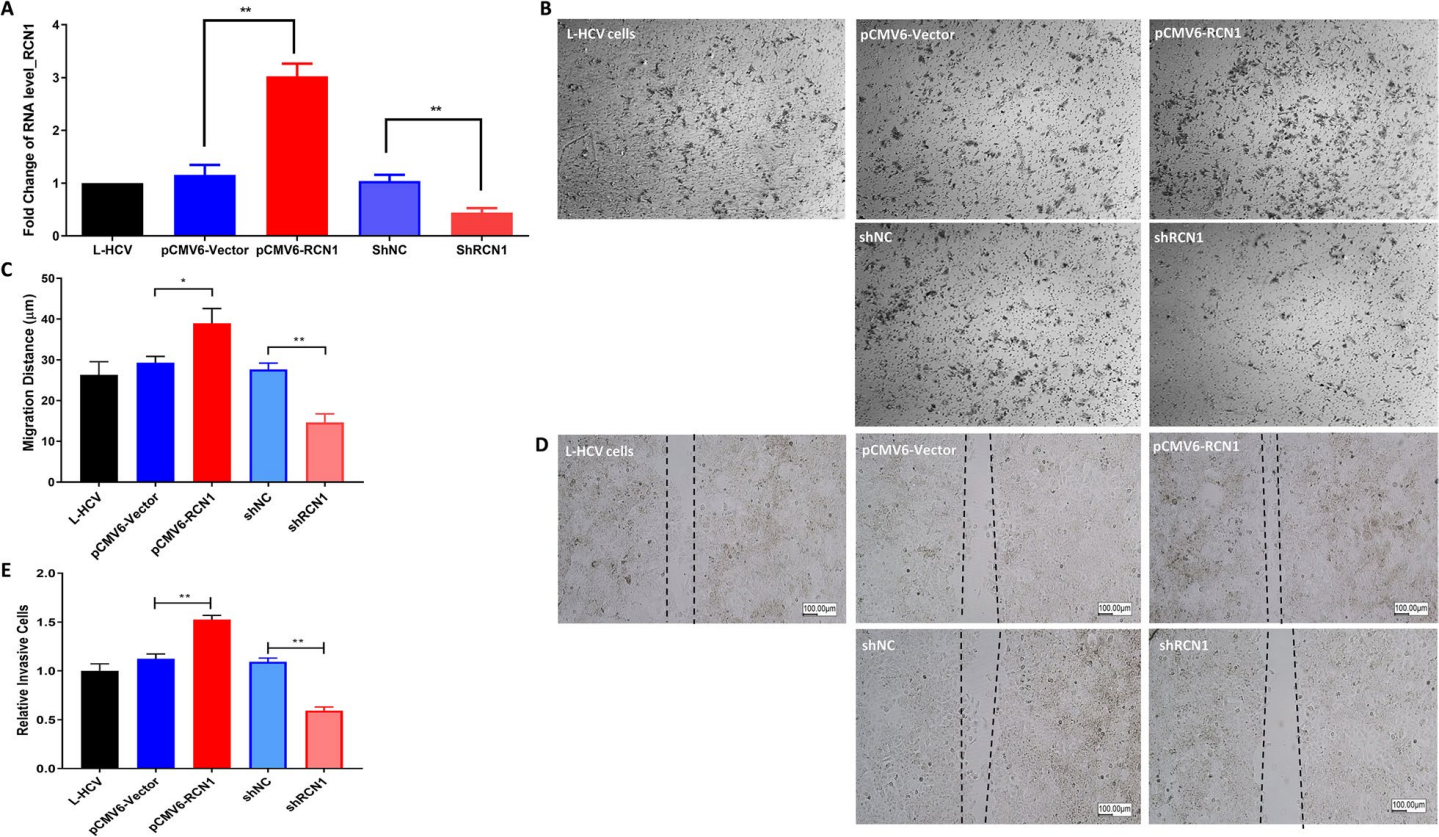
Invasion and migration assay of regulated RCN1 in L-HCV in vitro. **A** Expression of RCN1 in L-HCV cells transfected with pCMV6 empty vector, pCMV6-RCN1 (overexpressing RCN1), shNC (short hairpin normal control), and shRCN1 (short hairpin RCN1). Representative images and graphical display of cell invasion in L-HCV cells in vitro transwell assay: **B**, **C** Quantitative and qualitative analysis of L-HCV cells transfected with pCMV6 empty vector, pCMV6-RCN1, shNC, and shRCN1. **D**, **E** Quantitative and qualitative analysis of L-HCV cell migration in an in vitro wound healing assay in regulated RCN1. Time-lapse microscopy images of wound closure in L-HCV cells. Experiments were conducted in triplicates. Statistical significance p < 0.05*, p < 0.01**

To evaluate the role of RCN1 in EMT features, including cell invasion and migration, we performed an invasion assay and wound healing test by regulating RCN1 in JFH1-infected cells. Overexpression and silencing of RCN1 was confirmed (Fig. 4A). Figure 4B indicates that the Boyden chamber assay invasion capability of L-HCV increased significantly (p = 0.0004) due to overexpression of RCN1 (pCMV6-RCN1) (Fig. 4B and C). In contrast, silencing RCN1 (pCMV6-RCN1) significantly (p < 0.0001) inhibited invasion (Fig. 4B and C). The expression of RCN1 regulated invasion in JFH1-infected cells.

In the wound healing test, JFH1-infected cells were cultured for 48 h around a 500 μm wound insert. The width of the wound was significantly (p = 0.013) decreased in JFH1-infected cells transinfected with overexpressed RCN1 (pCMV6-RCN1) compared to the control group (Fig. 4D and E). In contrast, silencing RCN1 (shRCN1) led to significant (p = 0.006) inhibition of wound healing (Fig. 4D and E). Motility was significantly decreased in RCN1-suppressed JFH1-infected cells. The results showed that RCN1 regulated migration.

Collectively, combining RCN1 expression (downregulated) in the HCV-HCC cohort and miRNA (upregulated) expression suggests that miRNAs suppressed the expression of RCN1, which promoted invasion and migration in HCC. Therefore, RCN1 acts as a tumor suppressor in HCV-HCC. The Graphical abstract summarizes our findings.

## Discussion

MiRNAs play a crucial role in gene expression and regulation. Dysregulation of miRNA is linked to viruses such as HCV and diseases such as HCC [30]. However, there is still limited research on the regulatory relationship between miRNAs and mRNAs in persistent HCV-HCC. This study aimed to investigate the function and effects of persistent HCV-induced miRNA expression on gene regulation in HCC. Our findings showed that HCV infection induced the expression of miRNAs associated with survival in LIHC. These miRNAs regulate genes involved in viral carcinogenesis and proteoglycans pathways, associated with LIHC. These targeted genes in response to stimuli such as HCV alter the extracellular matrix through fibrillar collagen to regulate liver damage and fibrosis in HCC. HCV miRNAs target RCN1 to regulate EMT in HCV-HCC, thereby acting as a tumor suppressor by inhibiting invasion and migration in HCV-HCC.

MiRNAs are believed to negatively regulate gene expression by silencing target mRNA, and increasing evidence indicates that miRNAs alternate between repression and stimulation as a response to specific cellular conditions, sequences, and cofactors [31, 32]. Recent evidence suggests that different miRNAs can alter gene expression in certain cell types or conditions with distinct transcripts and proteins [33]. A single specific gene could encounter both regulation directions based on the specific conditions and factors such as HCV, LIHC or stress. Evidence demonstrate miRNAs as major regulators of HCV infection including HCV replication and distinguishing chronic HCV patients, and liver disease progression such as HCC [34, 35]. MiRNAs, especially miRNA clusters, have high diagnostic value for HCV-HCC [36]. Dysregulation of miRNAs due to HCV infection occurs via multiple pathways, such as the immune response, lipid metabolism, and cell cycle pathways [37]. A cluster of microRNAs is prominently expressed in primary liver tumors, where they serve as either tumor suppressors or oncogenic regulators [38]. MiRNA expression profiling shows abundantly upregulated miRNAs in HCC compared to patients with advanced fibrosis [39]. The diverse and complex mechanisms by which miRNAs regulate gene expression at both the cytoplasmic and nuclear levels suggest a consistent miRNA regulatory mechanism that alters cellular functions [40]. Host miRNAs are involved in different biological cycles of HCV, such as infection and replication. HCV infection regulates the expression of many cellular miRNAs involved in liver fibrosis, hepatocarcinogenesis and HCC progression [30]. Chronic HCV infection is a key cause of liver diseases leading to fibrosis and HCC [41]. Liver fibrosis is characterized by increased accumulation of constituents of the hepatic extracellular matrix, including collagen and elastin, which alters the microenvironment and favors cancer initiation and growth [41]. Furthermore, persistence in HCV infections leads to chronic inflammation, oxidative stress, and alterations in cellular signaling and metabolism [41]. HCV replication promotes the deposition of extracellular matrix (ECM) and accumulation in liver fibrosis [42]. Hepatocytes and biliary epithelial cells undergo an epithelial to mesenchymal transition, similarly assuming a fibrogenic phenotype [43].

HCV induces EMT in primary human hepatocytes [44]. HCV-infected hepatocytes displayed a fibroblast-like shape and an extended life span [44]. Increased mRNA and protein expression levels of EMT markers such as vimentin are observed in HCV-infected liver biopsy specimens, suggesting an onset of active EMT [44]. VIM has been identified as a powerful prognostic EMT marker in HCV-HCC for the prediction of recurrence [45]. The HCV protein induces EMT progression, which is part of understanding the mechanism of HCV-HCC development, invasion and metastasis [46]. All tumors and cancers present unique signatures of altered miRNA expression. MiR-10b is highly expressed in nasopharyngeal carcinoma cells, where it downregulates the expression of epithelial cell markers and alters the expression of mesenchymal cell markers such as vimentin (VIM) [47]. RCN1 plays an important role in a variety of solid tumors, including renal, breast, lung and liver cancer, where it regulates tumor invasion and migration [48–51]. Based on our findings in HCV-HCC cohort where RCN1 is downregulated, RCN1 regulates tumors as suppression of RCN1 inhibited invasion and migration. Suppression of RCN1 facilitates apoptosis and necroptosis in cancer cells [52]. Furthermore, RCN1 induces sorafenib resistance and malignancy in HCC by activating cancer signaling pathways [26]. In invasive cancer cells, RCN1 is overexpressed compared to poorly invasive cancer cells [49]. In prostate cancer, RCN1 expression increases in the presence of tumor necrosis factor and proinflammatory cytokines in advanced metastasis cancer patients [53]. MiR-215 and EMT-specific markers have been noted in different stages of chronic HCV infection [54]. EMT transcription and tumor metastasis is inhibited by miR-10b, miR-192-5p (miR‑215‑5p) and let-7a through different EMT-associated pathways and HCC hallmarks, leading to suppression of tumor viability in HCC [55–60].

MiR‑192‑5p and let-7a suppress the initiation and progression of cancer by repressing cell proliferation, decreasing the efficiency of sphere formation in stem-like cells to suppress tumor formation in HCC [59–62]. Overexpression of miR‑192‑5p and let-7a inhibit sphere formation and growth by inhibiting cell proliferation, migration and invasion of cancer [60–63]. Furthermore, increased expression of let-7a-5p leads to the inhibition of gene expression delaying HCC progression in vitro [64].

Finally, we believe that the candidate miRNAs identified within the panel provided an insightful regulatory relationship between mRNA in HCV-HCC. miR-215, miR-10b, and RCN1 could act as markers for HCV-HCC progression. RCN1 is proposed to play a significant role in the prognosis of HCV-HCC via inhibition of tumor invasion and migration, suggesting a potential prognostic biomarker ability for this gene. Thus, using the miRNA and gene combined panel will facilitate and improve HCV-HCC prognosis more than a conventional single biomarker approach.

## Limitations

There were a few limitations in our study; however, we believe that these limitations are not significant in altering our findings. We identified miRNAs based on their effect on survival outcome in LIHC, these miRNAs may have deviation in expression as some transcription factors may regulate alternating functions during HCV-HCC progression. MiRNAs can also bind multiple promoter regions of positively correlated genes. Hence, there is a constant need for prediction tools to be validated and verified. However, we were able to illustrate their effects on gene regulation and effects on HCC. Furthermore, genes are expressed simultaneously with miRNAs that may be upstream or downstream regulators that may be omitted during filtering. However, we were able to elucidate the miRNA mechanisms in gene regulation regarding HCV-HCC. The integration of HCV-HCC cohort data integrates genes directly associated with HCV-HCC allowed for a comprehensive understanding and identification of biomarkers of HCV-HCC that may be applied in a clinical setting.

Future research will involve the validation of the regulation of miRNA candidates and their target RCN1 in an animal model to understand the relationship between HCV-HCC and biochemical factors which would further eliminate risk factors associated with lifestyle habits and healthy related issues. The miRNA expression profile in HCV and its target RCN1 may be potential biomarkers for the diagnosis of HCV-HCC and the miRNAs even applied in therapeutics to regulate gene expression. Therefore, miRNAs may represent a promising non-invasive class of molecular biomarkers.

In conclusion, our study identified novel prognostic markers and the functional roles of prognostic miRNAs and genes in HCV-HCC. The dysregulation mechanism and effects of miRNA on gene regulation were highlighted. Thus, our findings have provided several biomarkers for the diagnosis and progression of HCV-HCC patients, as well as potential targeted therapy for HCV-HCC patients.

### Supplementary Information

**Additional file 1: Figure S1.** MiRNA expression in LIHC and HCV-JFH1 infectious cells. S-HCV miRNAs expression between cancer and normal samples in LIHC clinical database: (A) hsa-miR-21-5p, (B) hsa-miR-4286, (C) hsa-miR-939-5p, (D) hsa-miR-151a-5p, and (E) hsa-miR-29b-3p. L-HCV miRNAs expression between cancer and normal samples in LIHC clinical database: (F) hsa-miR-215-5p, (G) hsa-miR-10b-5p, (H) hsa-miR-455-3p, (I) hsa-miR-6717-5p, (J) hsa-miR-483-5p, and (K) hsa-let-7a-5p. Statistical significance P<0.01. (L) MiRNA relative normalized data ratio expression between S-HCV and L-HCV.

**Additional file 2: Figure S2.** Survival plots of the five combined L-HCV LIHC significant miRNAs in different cancers. : (A) OV - Ovarian Serous Cystadenocarcinoma (N=484), (B) EASCA - Esophageal Carcinoma (N=184), (C) COAD - Colon Adenocarcinoma (N= 424), (D) LUAD – Lung Adenocarcinoma (N= 500), (E) PAAD- Pancreatic Adenocarcinoma (N=177). (F) PRAD – Prostate Adenocarcinoma (N=494). Statistical significance p < 0.05.

**Additional file 3: Figure S3.** Kaplan-Meier Plotter of hepatitis associated liver cancer disease specific survival genes. Significantly disease specific survival (DSS) in hepatitis related liver cancer: (A) RCN1, (B) MMP11, (C) COL14A1, (D) SLC38A9, (E) DKK3, (F) TTLL4, (G) SURF4, (H) TFRC, (I) BCL2L11, (J) PEG10, (K) MEF2C, (L) SYT7, (M) HK2, (N) TTL, (O) RDX, and (P) MSN.

**Additional file 4: Figure S4.** miRNA-mRNA binding sites. (A, B) Hsa-let-7a-5p binding sites on RCN1. (C, D) Hsa-miR-10b-5p binding sites on RCN1. (E, F) hsa-miR-215-5p binding sites on RCN1.

## Data Availability

The dataset(s) supporting the conclusions of this article is(are) available in the Gene Expression Omnibus (GEO) repository, GSE235959 and hyperlink to dataset(s) in https://www.ncbi.nlm.nih.gov/geo/query/acc.cgi?acc=GSE235959 for miRNA and GSE154211and hyperlink to dataset(s) in https://www.ncbi.nlm.nih.gov/geo/query/acc.cgi?acc=GSE154211 for HCV-HCC cohort.

## Abbreviations

miRNA: MicroRNA
HCV: Hepatitis C virus
GO: Gene Ontology
IPA: Ingenuity Pathways Analysis
L-HCV: Long-term HCV
S-HCV: Short-term HCV
Huh7.5.1: Human hepatoma 7.51
FACS: Fluorescence-Activated Cell Sorting
ENCORI: Encyclopedia of RNA Interactomes
BP: Biological process
MF: Molecular Function
CC: Cellular Compartment
KEGG: Kyoto Encyclopedia of Genes and Genomes
LIHC: Liver Hepatocellular Carcinoma
HR: Hazard Ratio
DE: Differentially Expressed
OV: Ovarian Serous Systadenocarcinoma
EASCA: Esophageal Carcinoma
COAD: Colon Adenocarcinoma
LUAD: Lung Adenocarcinoma
PAAD: Pancreatic Adenocarcinoma
PRAD: Prostate Adenocarcinoma
NGS: Next Generation Sequencing
EMT: Epithelial Mesenchymal Transition
RCN1: Reticulocalbin 1
ICC: Immunocytochemistry
VIM: Vimentin
DAPI: 4′,6-Diamidino-2-phenylindole
