# Supplementary figures and images for: Systematic integration of molecular and clinical approaches in HCV-induced hepatocellular carcinoma
Source: J Transl Med. 2024 Mar 12;22:268. doi: 10.1186/s12967-024-04925-1 (PMC10935926; doi:10.1186/s12967-024-04925-1)

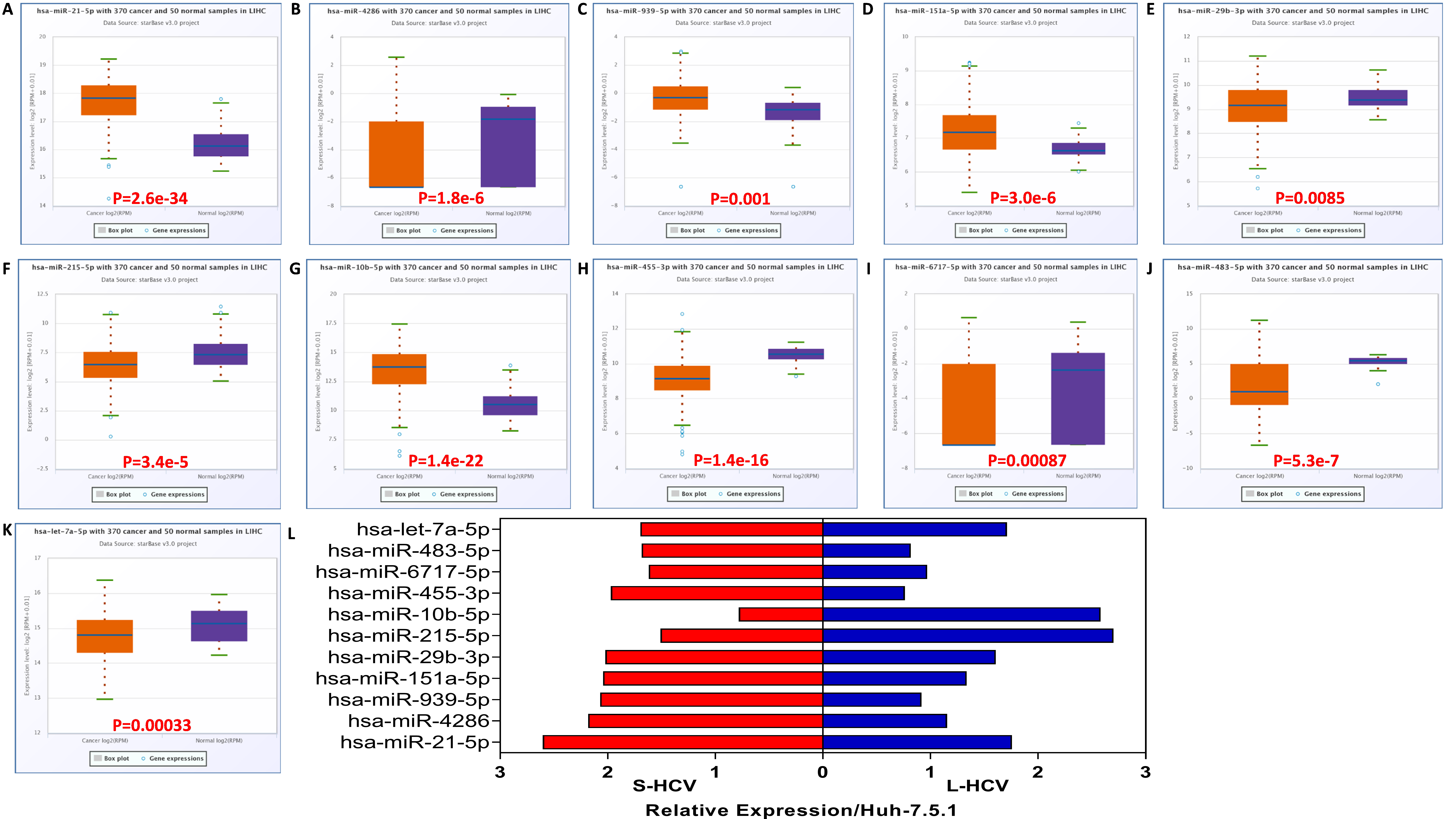

Supplement: Supplementary file 1 — Additional file 1: Figure S1. MiRNA expression in LIHC and HCV-JFH1 infectious cells. S-HCV miRNAs expression between cancer and normal samples in LIHC clinical database: (A) hsa-miR-21-5p, (B) hsa-miR-4286, (C) hsa-miR-939-5p, (D) hsa-miR-151a-5p, and (E) hsa-miR-29b-3p. L-HCV miRNAs expression between cancer and normal samples in LIHC clinical database: (F) hsa-miR-215-5p, (G) hsa-miR-10b-5p, (H) hsa-miR-455-3p, (I) hsa-miR-6717-5p, (J) hsa-miR-483-5p, and (K) hsa-let-7a-5p. Statistical significance P<0.01. (L) MiRNA relative normalized data ratio expression between S-HCV and L-HCV. [file 12967_2024_4925_MOESM1_ESM.tif]

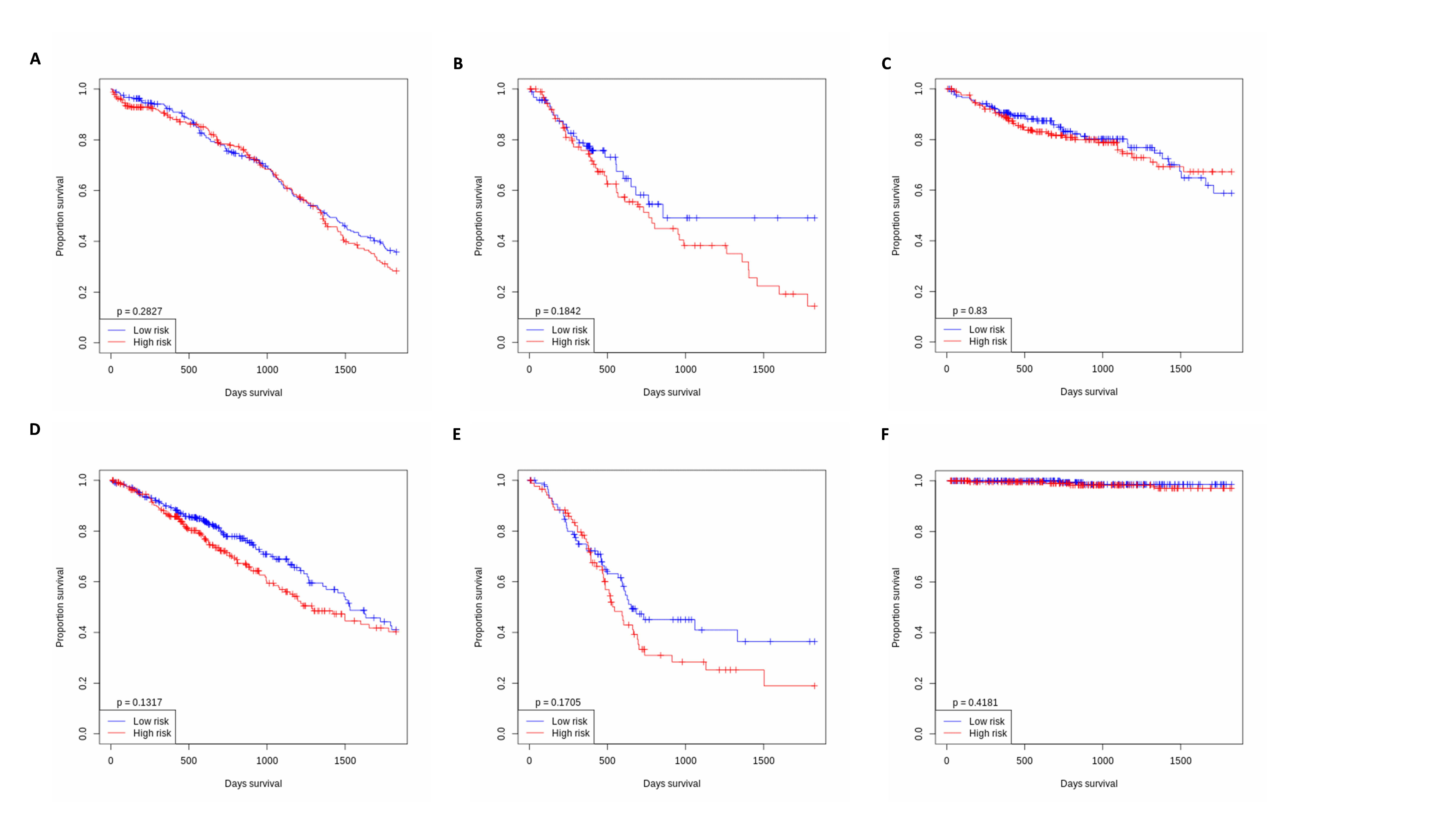

Supplement: Supplementary file 2 — Additional file 2: Figure S2. Survival plots of the five combined L-HCV LIHC significant miRNAs in different cancers. : (A) OV - Ovarian Serous Cystadenocarcinoma (N=484), (B) EASCA - Esophageal Carcinoma (N=184), (C) COAD - Colon Adenocarcinoma (N= 424), (D) LUAD – Lung Adenocarcinoma (N= 500), (E) PAAD- Pancreatic Adenocarcinoma (N=177). (F) PRAD – Prostate Adenocarcinoma (N=494). Statistical significance p < 0.05. [file 12967_2024_4925_MOESM2_ESM.tif]

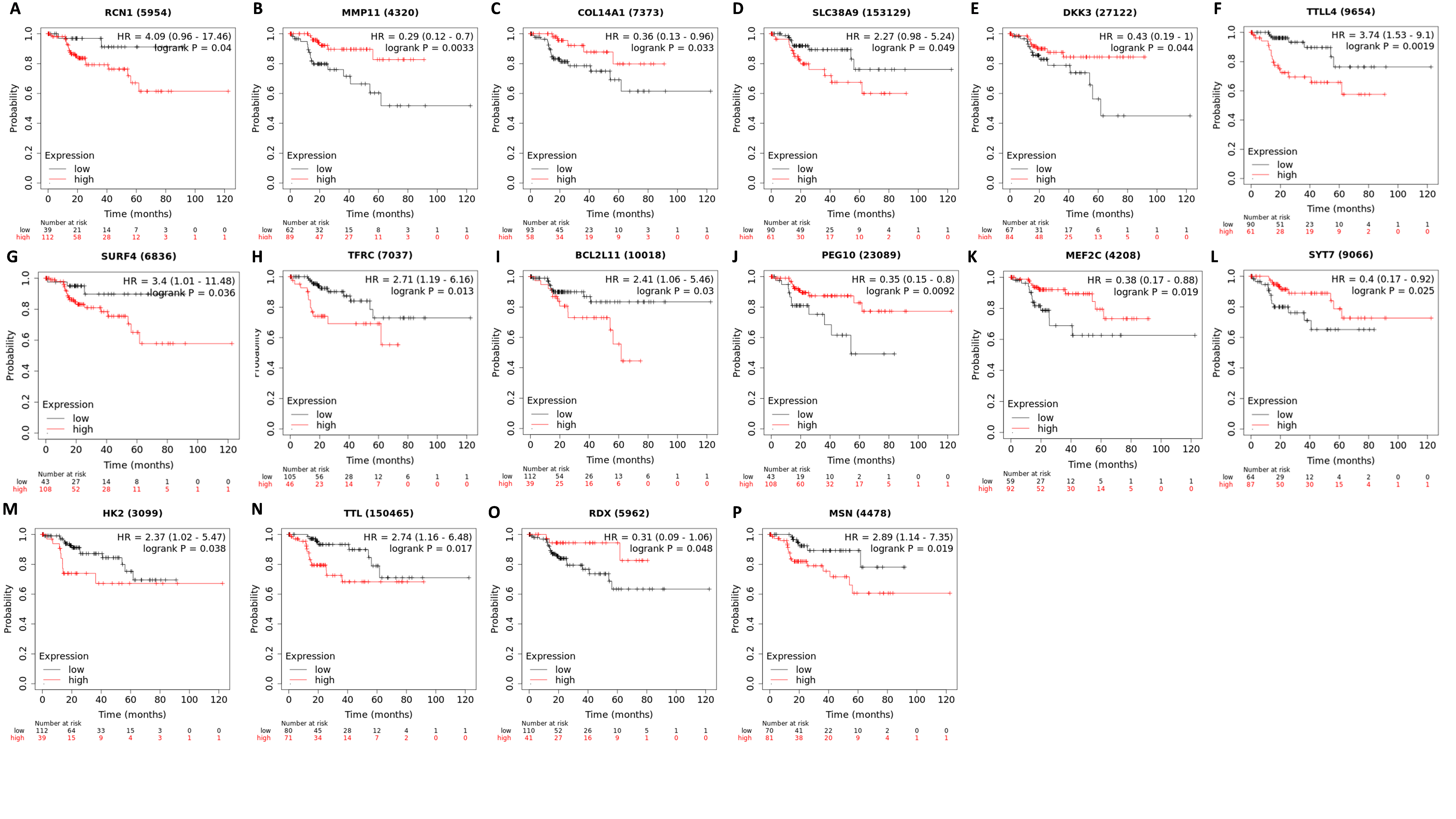

Supplement: Supplementary file 3 — Additional file 3: Figure S3. Kaplan-Meier Plotter of hepatitis associated liver cancer disease specific survival genes. Significantly disease specific survival (DSS) in hepatitis related liver cancer: (A) RCN1, (B) MMP11, (C) COL14A1, (D) SLC38A9, (E) DKK3, (F) TTLL4, (G) SURF4, (H) TFRC, (I) BCL2L11, (J) PEG10, (K) MEF2C, (L) SYT7, (M) HK2, (N) TTL, (O) RDX, and (P) MSN. [file 12967_2024_4925_MOESM3_ESM.tif]

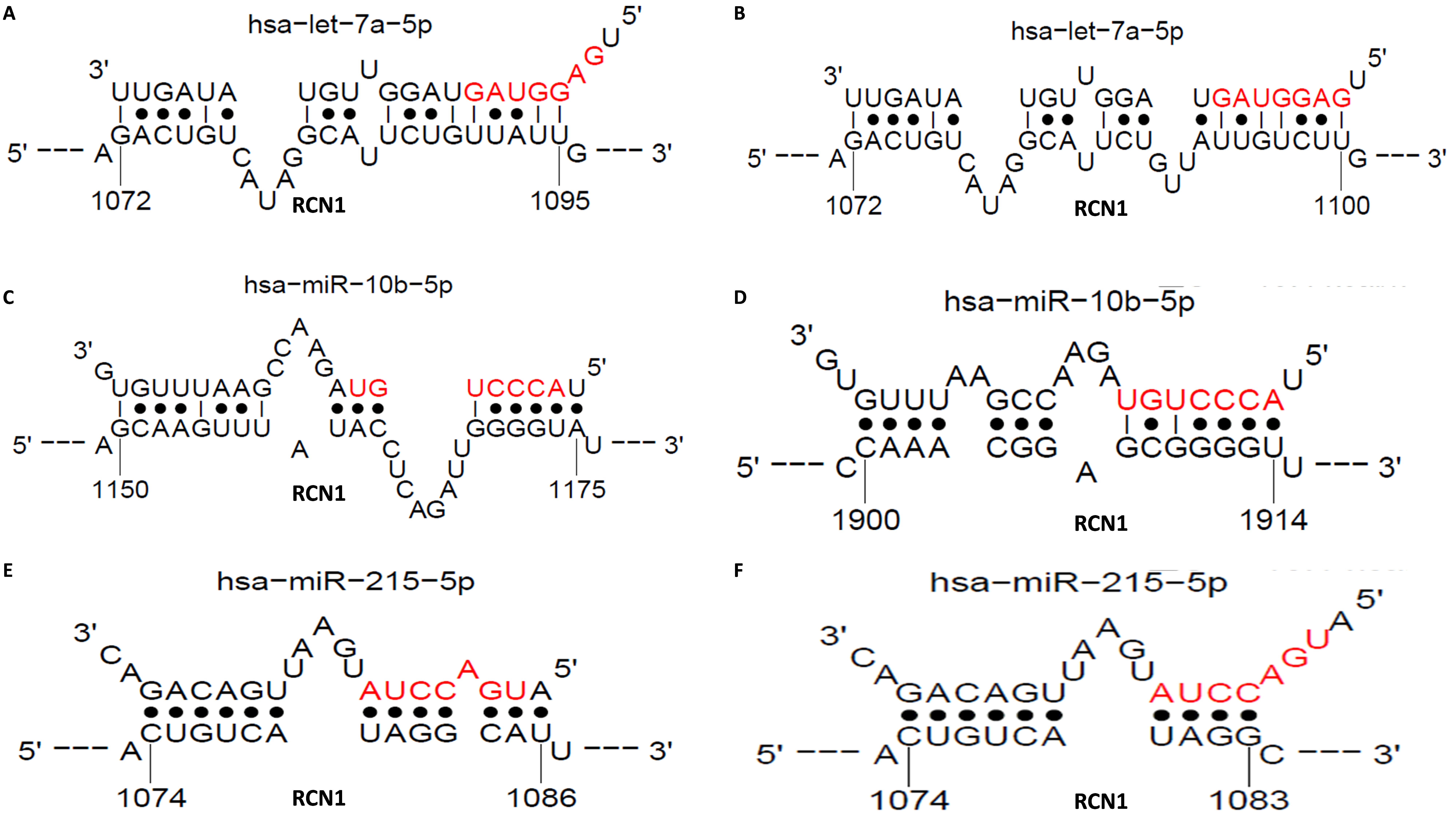

Supplement: Supplementary file 4 — Additional file 4: Figure S4. miRNA-mRNA binding sites. (A, B) Hsa-let-7a-5p binding sites on RCN1. (C, D) Hsa-miR-10b-5p binding sites on RCN1. (E, F) hsa-miR-215-5p binding sites on RCN1. [file 12967_2024_4925_MOESM4_ESM.tif]
